# Novel approach to incorporate information about recessive lethal genes increases the accuracy of genomic prediction for mortality traits

**DOI:** 10.1038/s41437-020-0329-5

**Published:** 2020-06-12

**Authors:** Grum Gebreyesus, Goutam Sahana, A. Christian Sørensen, Mogens S. Lund, Guosheng Su

**Affiliations:** Center for Quantitative Genetics and Genomics, Aarhus University, Blichers Alle, 8830 Tjele, Denmark

**Keywords:** Animal breeding, Genetic markers, Heritable quantitative trait, Population genetics

## Abstract

The genetic underpinnings of calf mortality can be partly polygenic and partly due to deleterious effects of recessive lethal alleles. Prediction of the genetic merits of selection candidates should thus take into account both genetic components contributing to calf mortality. However, simultaneously modeling polygenic risk and recessive lethal allele effects in genomic prediction is challenging due to effects that behave differently. In this study, we present a novel approach where mortality risk probabilities from polygenic and lethal allele components are predicted separately to compute the total risk probability of an individual for its future offspring as a basis for selection. We present methods for transforming genomic estimated breeding values of polygenic effect into risk probabilities using normal density and cumulative distribution functions and show computations of risk probability from recessive lethal alleles given sire genotypes and population recessive allele frequencies. Simulated data were used to test the novel approach as implemented in probit, logit, and linear models. In the simulation study, the accuracy of predicted risk probabilities was computed as the correlation between predicted mortality probabilities and observed calf mortality for validation sires. The results indicate that our novel approach can greatly increase the accuracy of selection for mortality traits compared with the accuracy of predictions obtained without distinguishing polygenic and lethal gene effects.

## Introduction

Important fractions of deleterious mutations segregating in diploid organisms are recessive alleles that cause fatal effects when present in a homozygous state. In dairy cattle breeds, intensive use of a limited number of elite breeding sires through artificial insemination has led to the spread of recessive lethal alleles in populations (e.g., Shuster et al. 1992; Agerholm et al. 2001). Recessive lethal alleles are of considerable economic consequence in the dairy industry because they cause calf and young stock mortality as well as reproductive inefficiency (e.g., Cole et al. 2016). Calf mortality represents a major economic loss for farmers, poses great animal welfare issues, and threatens public perceptions of the dairy industry. Therefore, efficient strategies for limiting the impact of recessive lethal alleles on calf mortality are critical.

Several strategies have been proposed that involve the use of genotype information to limit the harmful effects of recessive lethal alleles (e.g., Charlier et al. 2008; Pryce et al. 2012; Cole 2015), including the possibility of their removal through genome editing (Johnsson et al. 2019). Currently, the most practiced approach in dairy breeding is to limit carrier-to-carrier mating (Charlier et al. 2008). While such an approach might be feasible with few lethal alleles segregating in the population, it is becoming considerably more difficult with the increasing number of detected recessive lethal alleles (VanRaden et al. 2011; Fritz et al. 2013; Sahana et al. 2013, 2016; Kadri et al. 2014; Hoff et al. 2017). Preselection based on carrier status reduces selection intensity, consequently affecting genetic gains in economically important traits. In addition, the genetic causes of calf mortality are also partly polygenic. Therefore, genomic prediction of breeding values for calf mortality traits should take into account both polygenic and recessive lethal components.

Several approaches have been proposed for incorporating genotypic information of major genes into genomic prediction for traits controlled by both “major” genes and polygenic inheritance (e.g., Hoeschele 1988; Fernando and Grossman 1989). However, the proposed models traditionally rely on the assumption of additive effects for major quantitative trait loci (QTLs), as in the remaining genome-wide markers (Cole 2015), and different weights, with the a priori assumption of different effect sizes. Adopting such approaches to model polygenic effects and recessive lethal alleles simultaneously is problematic, as not only the effect sizes but also the modes of gene action are different. The effect of the heterozygous genotype for a recessive lethal locus on an individual is the same as that of the homozygous genotype of the wild type, but the effects on the future mortality risk of offspring differ. Additional sources of complexity in modeling the two effects simultaneously are that different recessive lethal alleles might have different penetrance levels and the fact that the mortality risk transferred to offspring depends on both penetrance and recessive allele frequency. Therefore, fitting polygenic and recessive lethal effects simultaneously in genomic prediction models has been a challenge.

In this study, we hypothesize that modeling risk probabilities from polygenic QTL effects and recessive lethal alleles separately can improve the accuracy of selection for mortality traits. The objective of this study is therefore to present a strategy where a breeding animal’s risk with regard to the mortality of its future offspring can be predicted in terms of probabilities for the recessive lethal allele and polygenic risk components. In this approach, risk probabilities from recessive lethal loci are computed as a function of carrier status and the recessive allele frequency in the population. We develop methodologies for transforming genomic estimated breeding values (GEBVs) into polygenic risk probabilities using normal density and cumulative distribution functions. Simulated data are used to test the advantages of the presented approach by comparing its prediction accuracies with those of an alternative approach where risk probabilities are predicted without distinguishing polygenic and recessive lethal allele effects.

## Materials and methods

In this section, we present an approach that allows the prediction of genetic risk posed to the survival of future offspring by both the recessive lethal locus and polygenic components of a breeding animal as the basis for selection to improve mortality traits.

### Risk probabilities from recessive lethal alleles

The computation of risk probabilities from recessive lethal alleles is straightforward when using the carrier status of an animal as inferred from its genotype and the recessive allele frequencies in the population. Given the genotype of a breeding animal, e.g., a sire, for a particular recessive lethal locus, an offspring’s risk probability of succumbing to a lethal gene is the probability that it receives two copies of the recessive lethal allele. Assuming a locus *i* with recessive lethal allele a and wild-type allele A, the probability of the offspring’s mortality due to the lethal allele (hereafter referred to as *p*_lethi_) is the probability of the offspring having an aa genotype. This can be computed given the sire’s genotype and the frequency of the recessive allele in the population (*p*_*a*_), which accounts for the probability of receiving a copy of the lethal allele from a dam under the assumption of random mating:

1 $$p_{\left( {{\rm{leth}}_i} \right)} = p\left( {{\mathrm{a}}\,{\mathrm{from}}\,{\mathrm{sire}}\,\left| {{{{\rm sire}\_{\rm geno}}}} \right.} \right) \times p_a.$$

Assuming complete penetrance and given three possible outcomes of the sire’s genotype, i.e., AA, Aa, or aa, the lethal risk probability at locus *i* for an offspring can be given as:

$${\rm{Sire}}\,{\rm{AA}}:\,p\left( {{\rm{leth}}_i} \right) = 0,$$

$${\rm{Sire}}\,{\rm{A}}{\mathrm{a}}:p\left( {{\rm{leth}}_i} \right) = 0.5 \times p_a,$$

${\rm{Sire}}\,{\rm{aa}}:\,p\left( {{\rm{leth}}_i} \right) = p_a$ (however, the affected individual may not be a breeding animal under complete penetrance).

In cases of recessive lethal alleles with a penetrance other than 100%, the risk probability in Eq. (1) can be calculated by multiplying by the penetrance level of a given lethal allele:

2 $$\begin{array}{l}p\left( {{\rm{leth}}_i} \right) = p\left( {{\mathrm{a}}\,{\mathrm{from}}\,{\mathrm{sire}}\,\left| {{{{\rm sire}\_{\rm geno}}}} \right.} \right) \times p_{a} \times {\rm{penetrance}}\end{array}.$$

An individual might carry more than one recessive lethal gene. Thus, the risk probability across all *n* loci can be computed as:

3 $$p\left( {{\rm{leth}}} \right) = 1 - \mathop {\prod}\nolimits_{i = 1}^n {\left( {1 - p\left( {{\rm{leth}}_i} \right)} \right)}.$$

### Prediction of polygenic risk probabilities

Obtaining risk probabilities from the polygenic component requires a transformation from GEBVs, which are predicted using different genomic prediction models. Mortality-related traits are often recorded as categorical outcomes, which are usually binary and hence non-normally distributed. Commonly used approaches for modeling categorical traits include threshold-liability (probit) and logistic regression (logit) models. Despite their violations of normality assumptions, linear models (LM) have also been employed in many studies for genetic analysis of categorical traits (e.g., Rao and Xia 2000; Peñagaricano et al. 2011). To accommodate the specific computations in the prediction of GEBVs in these different models, we present strategies for transforming GEBVs into polygenic risk probabilities as implemented in probit model, logit model, and LM.

#### Liability threshold (probit) model

In genetic modeling of categorical traits, one of the most commonly used approaches is the threshold model (Wright 1934). In the threshold model, the observed categorical responses are assumed to be the outcome of an underlying, normally distributed latent variable, often termed the liability (*l*), in relation to a fixed threshold (*τ*). In the context of mortality traits, the categories of response are usually “survival,” denoted as event 0, or “death,” denoted as event 1, i.e., a binary response, during a given monitored period. Accordingly, the observed categorical outcomes (*y*) are linked to the underlying liability (*l*) such that:

4 $$y = \left\{ {\begin{array}{*{20}{c}} {1,\,{\rm{if}}\,l > \tau } \\ {0,\,{\rm{if}}\,l < \tau } \end{array}} \right..$$

Thus, the expected liability (*η*) is assumed to be a function of the predictors such that:

5 $${\mathbf{\upeta }} = {\mathbf{X}}{{\mathbf\beta }} + {\mathbf{Za}},$$

where **η** is the vector of all expectations *η*_*i*_, **β** is the vector of fixed effects, **a** is the vector of random additive genetic effects, and **X** and **Z** are the design matrices for the fixed and random effects, respectively. Thus, the true underlying liability (*l*) will be the expectation plus the residuals such that:

6 $${\boldsymbol{l}} = {\mathbf{\upeta}} + {\boldsymbol{e}},$$

where ***l*** is the vector of all *l*_*i*_ and ***e*** is the vector of random residuals. The underlying liability for each individual as a linear function of the linear predictors can thus be rewritten as:

$$l_i = \eta _{\boldsymbol{i}} + e_i.$$

Combining Eqs. (4) and (6):

7 $$y = \left\{ {\begin{array}{*{20}{c}} {1,\,{\rm{if}}\,\eta _i + e_i > \tau } \\ {0,\,{\rm{if}}\,\eta _i + e_i < \tau } \end{array}} \right..$$

Given *η*_*i*_ estimated from the data and an assumed value for the fixed threshold *τ*, the observed outcomes are conditional on the residuals as follows:

8 $$y = \left\{ {\begin{array}{*{20}{c}} {1,\,{\rm{if}}\,\,e_i > \tau - \eta _i} \\ {0,\,{\rm{if}}\,\,e_i < \tau - \eta _i} \end{array}} \right..$$

The probability of observing event 1 (mortality) given *η*_*i*_ and *τ* can then be estimated as:

9 $${p}\left( {{y}_{i} = 1{\mathrm{|}}\eta _{\boldsymbol{i}},{\uptau}} \right) = {p}\left( {{e}_{i} > \tau - \eta _{\boldsymbol{i}}} \right) = 1 - \Phi _e\left( {\tau - \eta _{\boldsymbol{i}}} \right),$$

where *Φ*_*e*_(.) is the cumulative density function with $e_i\sim N\left( {0,\,\sigma _{e}^2} \right)$, where $\sigma _{e}^2$ is residual variance. During implementation of the probit model, the threshold *τ* is commonly set to 0 as a convenient origin. Since the liability cannot be observed, the variation in the liability is scaled to be $\sigma _{e}^2 = 1.$ Thus:

10 $${p}\left( {{y}_{i} = 1\left| {\eta _{\it{i}},{\it{\uptau }}} \right.} \right) = 1 - \Phi _e\left( { - \eta _{\it{i}}} \right),$$

with *e*_*i*_ ~ *N*(0, 1).

Considering the simplest case, where the population mean is the only fixed effect in the threshold model, i.e., **X**_**i**_***b*** = 1μ, the probability of observing event 1 can be estimated as:

11 $${p}\left( {{y}_{i} = 1\left| {\widehat \mu ,\widehat {a_i}} \right.} \right) = \widehat {\pi _i} = 1 - \Phi _e\left( { - \left( {\widehat \mu + \widehat {a_i}} \right)} \right),$$

where $\widehat \mu$ is the predicted population mean and $\widehat a_i$ is the EBV of individual *i*.

#### Logit model

Alternatively, a logistic distribution can be assigned to the residuals, resulting in a model known as the logit model. In this model, it is assumed that the logit of an underlying probability (*π*_*i*_) is a function of the linear predictors:

12 $${\rm{logit}}\,\left( {\pi _i} \right) = {\rm{log}}\left( {\frac{{\pi _i}}{{1 - \pi _i}}} \right) = {\boldsymbol{x}}_{\boldsymbol{i}}{\boldsymbol{b}}_{\boldsymbol{i}} + {\boldsymbol{z}}_{\boldsymbol{i}}{\mathbf{a}}.$$

Again, assuming a simple scenario, where the only fixed effect in the model is the population mean, Eq. (12) can be rewritten as:

$${\rm{logit}}\,\left( {\pi _i} \right) = {\rm{log}}\left( {\frac{{\pi _i}}{{1 - \pi _i}}} \right) = \widehat \mu + \widehat {a_i}.$$

The underlying probability (*π*_*i*_) can then be estimated by the inverse logit transformation:

13 $$\pi _i = {\rm{logit}}^{ - 1}\left( {\widehat \mu + \widehat a_i} \right) = \frac{{{\rm{Exp}}\left( {\widehat \mu + \widehat {a_i}} \right)}}{{1 + {\rm{Exp}}\left( {\widehat \mu + \widehat {a_i}} \right)}}.$$

#### Linear models

In addition to the probit and logistic regression models, it is a common practice in routine genetic evaluation to fit categorical trait data using LM, treating the traits as normally distributed. It has been shown that the loss of power when using a linear Gaussian model for categorical traits is negligible compared with implementing logit or probit regression, despite the violations of assumptions of normality (e.g., Meijering and Gianola 1985). Here, we present an approach for transforming EBVs obtained from a LM into risk probabilities in a way comparable to the probit approach. An advantage of the probit approach is that the threshold is usually set to 0. However, in a LM, the threshold (*τ*) must be estimated. Here, we estimate an approximate *τ* based on the cumulative distribution function of the normal distribution.

Given a particular threshold (*τ*), the proportion of an event (*y*_*i*_ = 1) (hereafter *π*) is the proportion of the underlying liability (*l*) above the threshold, i.e.:

14 $$1 - \pi = \Phi \left( {\uptau} \right),$$

where *Φ*(*τ*) is the cumulative probability of the normal distribution *N*(*μ*, *σ*^2^). *τ* is unknown, but *π* can be estimated as the proportion of observed *y*_*i*_ = 1 events in the data. Thus, the unknown threshold (*τ*) can be estimated by inverse probability transformation as:

15 $$\tau = \Phi ^{ - 1}\left( {1 - \pi } \right).$$

As mentioned above, the LM treats the binary observations as variables with a normal distribution. To be consistent with the mean and variance of the distribution for binary observations, the normal distribution used to derive *τ* is assumed to be:

$${N}\left( {\pi ,\,\pi \times \left( {1 - \pi } \right)} \right).$$

Furthermore, risk probabilities can then be estimated by Eqs. (9)–(11), but with an approximated threshold *τ*, which is not set to 0 as in the liability threshold model. Thus:

16 $${p}\left( {{y}_{i} = 1\left| {\widehat \mu ,\widehat a,\tau } \right.} \right) = \widehat \pi _i = 1 - \Phi _e\left( {\tau - \left( {\widehat \mu + \widehat a_i} \right)} \right).$$

Finally, since we are interested in the polygenic risk transmitted from a breeding animal, e.g., a sire, which on average passes half of its breeding value to its future offspring, the polygenic risk probability transmitted is half the probability of the sire estimated using the models presented above. Thus:

17 $$p_{{{{\rm poly}\_{\rm offspring}}}} = 0.5 \times p_{{{{\rm poly}\_{\rm sire}}}}.$$

### Combining risk probabilities from the lethal allele and polygenic components

The risk probabilities computed separately from the recessive lethal alleles and the polygenic effect can finally be combined to give the total risk probability of an individual’s future offspring. Assuming that the probabilities of survival from the lethal allele and polygenic components are independent, the total risk probability (*p*_*total*_) can be given as:

18 $$p_{{\rm{total}}} = 1 - \left( {\left( {1 - p_{{\rm{leth}}}} \right) \times \left( {1 - p_{{\rm{poly}}}} \right)} \right),$$

where *p*_leth_ is the risk probability from the lethal component and *p*_poly_ is the risk probability from the polygenic component.

### Assessing the accuracy of predicted risk probabilities

It is possible to assess the accuracy of predicted total risk probabilities with a validation procedure, where EBVs for the polygenic effect of validation individuals are predicted without using observations from their offspring. The predicted EBVs are subsequently transformed into risk probabilities, which together with the risk from the lethal genes are used to compute the total risk probability. The accuracy of the predicted risk probabilities can then be computed as the correlation between the predicted total risk probabilities of the test individual’s offspring and the observed proportion of offspring mortality.

### Simulation experiments

We tested the proposed approach with a dataset of 15 replicates simulated using the stochastic simulation program ADAM (Pedersen et al. 2009). In each replicate, a population of animals was simulated for 4 years with overlapping generations and assuming no selection. In each year, 50 males were randomly selected to mate with 10,000 females of different parities, with each male mated to 200 females. Offspring’s sex was assigned randomly with a probability of 50% for males and females. The simulations resulted in a total of 40,000 individuals in four generations with approximately equal proportions of males and females. Animals born in generations 1–3 were used as the reference population, while animals in generation 4 (G4) were used as the test population.

Genotype data were simulated mimicking the real linkage disequilibrium profile in the Danish Holstein as described in detail by Thomasen et al. (2019). The simulated genotype data included 40K markers, 1980 QTLs with polygenic effects, and 20 lethal genes with recessive allele frequencies between 0.04 and 0.05. A large number of QTLs with polygenic effects were assumed in order to mimic a trait of mixed polygenic-major gene inheritance, where a large number of QTLs with small effects each and a few lethal genes composed the genetic architecture. Other simulation studies mimicking the bovine genome for genomic prediction have assumed similar number of QTLs (Lourenco et al. 2013), or slightly more QTLs (Hayes et al. 2009; Thomasen et al. 2019), underlying various polygenic traits. The assumed recessive allele frequencies were chosen based on the range reported for recessive lethal haplotypes detected in the Danish and Nordic cattle breeds (Wu et al. 2020). SNPs were distributed across 30 chromosomes, each 100 cM in length. The QTLs were assumed to be evenly distributed across the genome, such that on each chromosome, 66 SNPs were randomly sampled to be QTLs. The effect of each QTL was sampled from a normal distribution, and the effects collectively explained all the variation in the simulated polygenic true breeding values (TBV).

Thus, the TBV for each animal *i* was defined as the sum of all QTL genotypic values:

$${\mathrm{TBV}}_{i} = {\sum} {{g}_{j}\,{Q}_{{ij}}},$$

where *g*_*j*_ is the allele substitution effect of *j*th QTL, *Q*_*ij*_ is the QTL genotype at locus *j* in individual *i*, coded as 0, 1, or 2 representing the number of copies for a particular allele in the genotype. The TBVs were finally scaled to have a variance of 1 in base population through dividing TBV by the standard deviation of TBVs in the base population, i.e., setting the additive genetic variance as: $\sigma _a^2 = 1$.

Simulation of the TBVs was performed at the liability scale, with a target heritability of 0.02 at the observed scale, according to heritability estimates reported in the literature for the Holstein breed (e.g., Hansen et al. 2003; Fuerst-Waltl and Sørensen 2010; Henderson et al. 2011). The target observed-scale heritability was transformed to the underlying scale using the formula proposed by Dempster and Lerner (1950):

19 $$h_l^2 = \frac{{h_x^2 \times \pi \left( {1 - \pi } \right)}}{{z^2}},$$

where $h_l^2$ is the heritability at the underlying scale, *z* is the height of the normal distribution curve at the threshold, $h_x^2$ is the heritability at the observed scale, which is 0.02 in this study, and *π* is the proportion for *y* = 1 (*π* = 0.068 in this study). Thus, heritability at the underlying scale was 0.075.

Simulated QTLs were not included in the construction of the genomic relationship matrices (GRMs) used for prediction. Recessive lethal loci were assigned by randomly sampling from SNPs with minor allele frequencies (MAFs) between 0.04 and 0.05 on 20 randomly selected chromosomes. Each lethal locus was located on a different chromosome, and the loci were thus assumed to be independent of one another.

Four different scenarios were simulated with regard to the penetrance of recessive lethal alleles. These included three scenarios where all 20 lethal alleles were assumed to have an equal penetrance of 60, 80, or 100%. The fourth scenario considered a mixture of four penetrance groups (with an equal number of lethal alleles): 60, 70, 80, and 100% penetrance.

Liability for death was generated by adding a residual effect to TBV. The residual effect was sampled from *e* ~ *N*(0, $\sigma _e^2$), where the residual variance ($\sigma _e^2$) was $\frac{{\left( {1 - h_l^2} \right)\sigma _a^2}}{{h_l^2}}$ = (1 − 0.075)/0.075. Simulated phenotypic values in observed scale were either 0 if the animal survived or 1 if the animal died. Individuals were assigned phenotypic values in a stepwise manner, considering both polygenic and recessive lethal allele components. First, a threshold was calculated as the inverse cumulative distribution function of a target mortality incidence (*y* = 1) of 6.8% from a normal distribution with mean 0 and variance $\sigma _a^2 + \sigma _e^2$. Individuals with a liability greater than the threshold were subsequently assigned a phenotypic value of 1. Second, an individual’s phenotype was assigned as 1, regardless of the assigned phenotype due to polygenic component, if its genotype for at least one of the recessive lethal alleles was in a homozygous state. When recessive lethal alleles were assigned a penetrance other than 100%, a proportion of the homozygous individuals for each allele was assigned a phenotype of 1 in accordance with the penetrance value assumed (60, 80%, or a mixture with an average of 75%). The average observed mortality over 15 replicates was 9.43%. On average, 29.73% of the total mortality was caused by recessive lethal allele effects, while 68.21% was due to polygenic risk and 2.06% was due to both.

### Statistical analysis

Breeding values and risk probabilities were estimated using the above approach. To predict risks from polygenic and recessive lethal allele effects separately, the data used to predict polygenic effects excluded records of death caused by recessive lethal alleles (Data_poly). Similarly, the GRM used for the prediction of GEBVs was constructed without recessive lethal alleles. All GRMs used for the different scenarios were calculated using the first method presented by VanRaden (2008), and SNP allele frequencies for building GRMs were calculated directly from the SNP data. Risk probabilities from the recessive lethal alleles were computed separately and then used to compute the total risk using Eq. (18).

To test the proposed approach’s superiority over the conventional approach, breeding values and risk probabilities were also estimated using a conventional approach that did not distinguish the polygenic and lethal allele effects. Thus, the conventional approach predicted total breeding values using phenotypic data without excluding observations of death due to recessive lethal alleles (Data_all) and GRMs that did not include recessive lethal genotypes. Subsequently, the predicted GEBVs were transformed into total risk probabilities.

In addition, we also assessed the accuracy of the polygenic GEBVs predicted without distinguishing the two effects, i.e., with the data that included death due to recessive lethal alleles, but taking into account the effects of the recessive lethal alleles, either by including recessive lethal alleles in the construction of the GRM or using models that included fixed regression on recessive allelic genotype code. Below, we present the methods used to predict the GEBVs that were subsequently transformed into risk probabilities for each of the models (probit, logit, and linear).

These approaches were implemented using three statistical models, namely, generalized linear mixed models with logit and probit link functions and a linear mixed model, using DMU software (Madsen and Jensen 2013).

### The probit models for analysis of the simulated data

Three probit models were used to estimate breeding values and risk probabilities due to polygenic effects. The first probit model was:

20 $${\mathrm{Probit1}}:{\mathbf{\upeta}} = {\mathbf{1}}\mu + {\mathbf{Z}}{\mathbf{g}},$$

where element *i* of **η** is *η*_*i*_ = *Φ*^−1^(*π*_*i*_), *μ* is the overall mean, **g** is the random additive genetic effects with distribution ${\mathbf{g}}\sim N\left( {0{\mathrm{,}}\,{\mathbf{G}}{\it{\upsigma }}_{\it{a}}^2} \right)$, and **G** is the GRM constructed using only markers, i.e., excluding the recessive lethal loci. Both Data_poly and Data_all were analyzed using this model.

The second probit model includes fixed regression on lethal genotype:

21 $${\mathrm{Probit2:}}\,{\mathbf{\upeta}} = {\mathbf{1}}\mu + {\mathbf{x}}d + {\mathbf{Z}}{\mathbf{g}},$$

where *d* is the fixed regression coefficient for lethal genotype score and ***x*** is the vector of recessive lethal statuses. Since a homozygous recessive genotype at a given locus can cause mortality regardless of the genotype at another recessive lethal locus, the element of *x* is 1 as long as the recessive lethal allele is in a homozygous state at any locus and “0” otherwise. The G matrix in this model also excluded genotypes of recessive lethal loci, and this model was used to analyze Data_all.

The third probit model was implemented to test the impact of including recessive lethal alleles in the GRM on genomic prediction:

22 $${\mathrm{Probit3:}}\,{\mathbf{\upeta}} = {\mathbf{1}}\mu + {\mathbf{Z}}{\mathbf{g}}^ \ast ,$$

where g^*^ are the vectors of random additive genetic effects with distribution $N\left( {0,\,{\mathbf{G}}^ \ast \sigma _{{a}^ \ast }^2} \right)$, where G^*^ is the GRM based on the markers including lethal alleles and $\sigma _{{a}^ \ast }^2$ is the corresponding genetic variance. This model was used to analyze Data_all.

### The logit models for analysis of the simulated data

The logit models used in the analysis were:

23 $${\mathrm{Logit1:}}\,{\mathbf{\upeta}} = {\mathbf{1}}\mu + {\mathbf{Z}}{\mathbf{g}},$$

24 $${\mathrm{Logit2:}}\;{\mathbf{\upeta}} = {\mathbf{1}}\mu + {\boldsymbol{x}}{d} + {\boldsymbol{Z}}{\mathbf{g}},$$

25 $${\mathrm{Logit3:}}\;{\mathbf{\upeta}} = {\mathbf{1}}\mu + {\mathbf{Z}}{\mathbf{g}}^ \ast,$$

where element *i* of **η** is $\eta _i = {\rm{log}}\left( {\frac{{\pi _i}}{{1 - \pi _i}}} \right)$ and the rest of the model components are the same as those in Models (20)–(22).

### The linear models for analysis of the simulated data

The implemented LM included three genomic best linear unbiased prediction models:

26 $${\mathrm{LM1:}}\,{\mathbf{y}} = {\mathbf{1}}\mu + {\mathbf{Z}}{\mathbf{g}} + {\mathbf{e}},$$

27 $${\mathrm{LM2:}}\,{\mathbf{y}} = {\mathbf{1}}\mu + {\boldsymbol{x}}{d} + {\mathbf{Zg}} + {\mathbf{e}},$$

28 $${\mathrm{LM3:}}\,{\mathbf{y}} = {\mathbf{1}}\mu + {\mathbf{Zg}}^ \ast + {\mathbf{e}},$$

where **y** is the vector of observations (0, 1) and **e** is the vector of residuals with distribution ${\mathbf{e}}\sim {N}\left( {0,{\mathbf{I}}\,\sigma _e^2} \right),$ where **I** is an identity matrix and $\sigma _e^2$ is the residual variance. The rest of the model components in (26)–(28) are as described in Models (20)–(22).

### Computation of prediction accuracy and bias

To assess the accuracy of predicted risk probabilities, the predicted total transmitted risk probabilities of the sires of the G4 animals were compared with the observed proportion of deaths among their G4 offspring, whose phenotypes were masked during the prediction. This is to be consistent with the situation where the candidate animals do not have offspring at the time of selection. For the approach that distinguished polygenic and recessive lethal allele effects, the predicted total risk probability was calculated using Eq. (18) to combine the risk probabilities from the recessive lethal allele and polygenic components obtained from GEBVs that were predicted using LM1, Logit1, or Probit1 and Data_poly. For the analysis that did not consider lethal genes, the predicted total risk probabilities were calculated from the GEBVs predicted using LM1, Logit1, or Probit1 but based on Data_all. Prediction bias was measured as the coefficient of regression of the observed rate of calf mortality against the predicted probabilities for the validation sires.

In addition, the advantages of incorporating recessive lethal alleles, by either including them in the GRM (LM3, Logit3, and Probit3) or considering lethal genotype as a fixed effect in regression (LM2, Logit2, and Probit2) to predict polygenic breeding values, were assessed by the accuracy of GEBVs, which was measured as the correlation between GEBVs and simulated polygenic TBVs.

The statistical significance of differences in prediction accuracies between scenarios (approaches) and models, i.e., probit, logit, and linear, was tested using a pairwise *t*-test across the replicates.

## Results

### Accuracy and bias of predicted risk probabilities

**Fig. 1:**
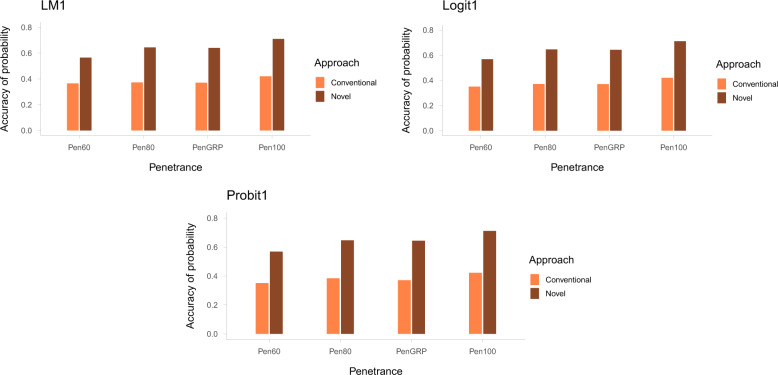
Risk probability prediction accuracies in the two approaches. Accuracies of predicted total risk probabilities obtained by the novel approach, distinguishing polygenic and recessive lethal allele effects (based on Data_poly), and conventional approach, not distinguishing polygenic and recessive lethal allele effects (based on Data_all), across the penetrance scenarios, plotted for each model (LM1, Probit1, and Logit1).

Figure 1 presents the accuracy of the total risk probability predicted with the two approaches in comparison: (1) the novel approach in which risk probabilities from the polygenic and recessive lethal components were estimated separately, with the polygenic component predicted using LM1, Logit1, or Probit1 based on Data_poly, and (2) the conventional approach where risk probability was estimated without distinguishing the polygenic and recessive lethal effects and obtained from GEBVs predicted using LM1, Logit1, or Probit1 but based on Data_all. Across all penetrance scenarios, the accuracies obtained with the novel approach were significantly higher (*P* < 0.001) than those obtained with the conventional approach. The difference in prediction accuracy between the two approaches ranged between 20 and 29.1 percentage points, depending on the penetrance scenario assumed.

In all three statistical models, i.e., LM1, Probit1, and Logit1, the highest accuracy was observed when all lethal alleles had 100% penetrance (Pen100), while the lowest accuracy was observed when all alleles had the lowest penetrance (Pen60). Among the three models, Probit1 resulted in the highest prediction accuracies, followed by Logit1, in both approaches. However, these differences in prediction accuracy between the three models were not statistically significant.

Table 1 presents the regression coefficients for observed calf mortality against predicted total risk probability for the novel approach which distinguished recessive lethal allele and polygenic effects and used Data_poly, and the conventional approach, which did not distinguish the two effects and used Data_all. For the analysis distinguishing the two effects, the regression coefficients were close to 1 for all three models and penetrance scenarios. For the analysis with conventional approach, however, the regression coefficients deviated from 1 for all models and penetrance scenarios, with the largest deviation observed for the LM.

**Table 1:** Regression coefficients for observed mortality against predicted total risk probability, obtained by the approach distinguishing polygenic and recessive lethal allele effects (based on Data_poly) and the approach not distinguishing polygenic and recessive lethal allele effects (based on Data_all).

Model	Data_poly				Data_all			
	Pen100	PenGRP	Pen80	Pen60	Pen100	PenGRP	Pen80	Pen60
LM1	1.086	1.086	1.100	1.125	2.073	1.989	2.061	2.195
Logit1	1.066	1.061	1.070	1.072	1.344	1.252	1.303	1.275
Probit1	1.072	1.069	1.080	1.089	1.412	1.332	1.428	1.362

Pen60, 80, 100, and GRP = penetrance level of 60, 80, 100 and a mixture of 60%, 70%, 80%, and 100% penetrance levels, respectively. Data_poly = phenotype data that excluded the records of death due to lethal alleles. Data_all = phenotype data that included the records of death due to lethal alleles.

### Accuracy of prediction of GEBVs

**Fig. 2:**
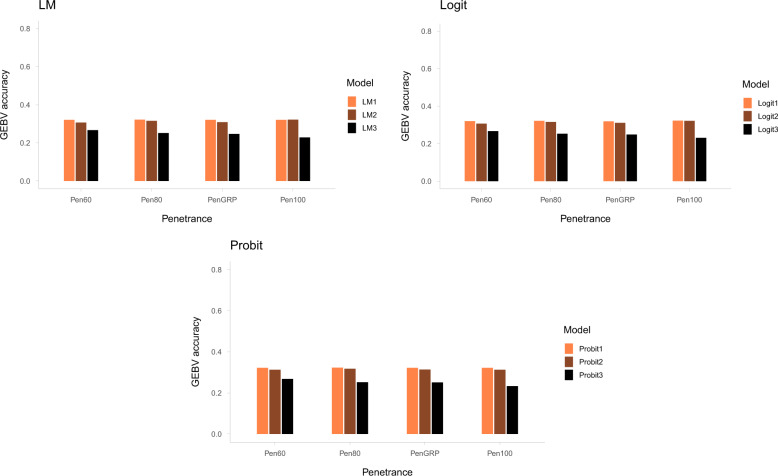
GEBV accuracies across the different approaches. Accuracy of GEBVs predicted using phenotypic data that excluded the records of mortality due to lethal alleles (Data_poly) with the LM1, Probit1, and Logit1 models; data that included records of mortality due to lethal alleles (Data_all) and models considering regression on lethal genotype (LM2, Probit2, and Logit2); and data that included records of mortality due to lethal alleles (Data_all) and models with a GRM including recessive lethal alleles (LM3, Probit3, and Logit3).

Figure 2 shows the accuracy of GEBVs predicted with the different approaches: (1) using Data_poly and a model with a GRM that did not include recessive lethal loci (LM1, Probit1, and Logit1), (2) using Data_all and a model with regression on lethal genotype (LM2, Probit2, and Logit2), and (3) using Data_all and a model with a GRM including genotypes of recessive lethal loci. In general, approach (1) resulted in the highest accuracy of predicted GEBVs, ranging from 0.319 to 0.323 according to penetrance class. Approach (2) resulted in slightly lower accuracies compared with those in approach (1), ranging from 0.307 to 0.322. However, the differences in GEBV prediction accuracies between the two approaches were only statistically significant in scenarios Pen60 (*P* < 0.01) and PenGRP (*P* < 0.05). Among the three approaches, approach (3) produced the lowest GEBV prediction accuracies. The prediction accuracies obtained using this approach were significantly lower (*P* < 0.001) than those obtained using approaches (1) and (2).

## Discussion

### Prediction of risk probabilities

Genomic prediction for traits with mixed “major” genes and polygenic inheritance has been shown to benefit from models that account for differences in marker effects compared with models with “infinitesimal” assumptions (e.g., Cole et al. 2009; Hayes et al. 2010; Legarra et al. 2011). Recessive lethal loci might be considered special cases of “major” genes. While a single recessive lethal locus might have a large effect on an observed phenotype, the effect on the individual itself might be different from the effect on its future offspring. The carrier status of a single recessive lethal allele alone does not affect the carrier’s mortality but can determine the categorical outcome (death or survival) of an offspring. In this study, we present an efficient approach for predicting the total risk probabilities for future offspring of selection candidates by predicting risk probabilities from polygenic and recessive lethal components separately. By using simulated data, the prediction accuracies of this approach were compared with those of a conventional approach that did not distinguish polygenic and recessive lethal allele effects (Data_all vs. Data_poly in Fig. 1). The results show that the prediction of risk probabilities with the proposed approach leads to high accuracy in predicting mortality for the future offspring of selection candidates, with a gain in accuracy up to 29.1 percentage points. By blending the risk probabilities estimated separately for the two components, the novel approach allows efficient utilization of information from the recessive lethal component, which otherwise tends to be difficult to untangle with simultaneous modeling due to differing modes of gene action. The gain in accuracy achieved by distinguishing the polygenic and recessive lethal allele effects, compared with the approach that does not distinguish the two effects, is dependent on the rate of mortality caused by the two effects. In our analysis, the gain in accuracy (29.1%) was comparable to the rate of mortality caused by recessive lethal alleles in the simulation (29.73%).

A potential challenge for the separate prediction of risk probabilities caused by polygenic and lethal allele effects in real data scenarios might be the difficulty of conclusively distinguishing mortality caused by recessive lethal alleles and that caused by polygenic components. This is because genotypic information may not be available for dead animals. In this study, we further investigated if taking into account the recessive lethal genotypes through, either including regression on lethal genotypes in the models or by accounting for genotypes of the recessive lethal loci in the GRM, could improve the accuracy of predicted GEBVs when using data that included mortality due to lethal alleles. The results showed that when the data included records of mortality caused by recessive lethal alleles, including the recessive lethal alleles in the GRM did not improve GEBV prediction accuracy. In contrast, when using data that included records of mortality due to lethal alleles, models with regression on lethal genotype resulted in prediction accuracy being comparable to the approach that distinguished the two effects using Data_poly. Compared with including genotypes of recessive lethal loci in the GRM, using models with fixed regression on lethal genotypes improved the accuracy of polygenic GEBVs by 4–9.3 percentage points, based on data that included records of mortality due to lethal alleles. These results demonstrate that in the situations where excluding mortality caused by recessive lethal alleles is difficult, using models with regression on lethal genotypes can improve prediction accuracies. Potential challenges in the approach considering fixed regressions on lethal genotype is the definition of a lethal covariable and the uncertain relationship between a lethal covariable and observations in the case of incomplete penetrance and unequal penetrances among lethal loci. Consequently, prediction accuracies might be affected by penetrance. This was demonstrated in our simulation, where the prediction accuracy of the model considering fixed regression on lethal genotype was significantly lower than that of the approach using Data_poly for scenarios having a lower penetrance (Pen60) and a mixture of penetrance levels (PenGRP).

In our simulation study, the accuracy of the predicted GEBVs was generally low across the compared models. This is expected given the low heritability considered in the simulation. In dairy cattle breeding, definitions of different calf and young stock mortality traits are dependent on monitoring period. In general, studies across several dairy cattle breeds have shown very low heritability estimates for calf and young stock mortality traits (e.g., Hansen et al. 2003; Fuerst-Waltl and Sørensen 2010; Henderson et al. 2011), which limit the expected genomic prediction accuracy. However, given the major deleterious effects of individual recessive lethal genes, the prediction accuracy for mortality traits can be improved with the efficient incorporation of genotypic information on such genes. The results of this study indicate that the presented novel approach is quite advantageous in integrating information on recessive lethal and polygenic components for the prediction of mortality traits. By bringing the two components to a comparable scale, i.e., risk probability, the approach allows utilizing information from both effects to predict the mortality status of future offspring of a breeding animal. A somewhat comparable approach to the prediction of risk probabilities presented in this study is the polygenic risk score (PRS) approach, which is commonly used in human genetics to predict an individual’s risk of succumbing to a particular disease (Wray et al. 2007, 2019; Evans et al. 2009). However, the PRS used in human genetics is predicted based on SNP effects estimated from genome-wide association studies that are often based on fitting one SNP at a time, thus ignoring all other SNPs (Wray et al. 2007). Moreover, PRSs in human genetics are used to predict the future phenotypes of an individual, while the primary objective in our approach, and in animal breeding more generally, is to predict a selection candidate’s transmission ability to its future offspring.

There are several assumptions in our simulation study that might not be fully consistent with the features of real data and thus might affect prediction accuracies to some extent. We have shown that the gain in accuracy achieved by distinguishing polygenic and recessive lethal allele effects is dependent on the rate of mortality caused by recessive lethal alleles. This rate, in turn, depends on the number of recessive lethal loci and recessive allele frequencies. In the simulation, 20 loci with lethal allele frequencies between 0.04 and 0.05 were assumed. These might be considered high-frequency lethal alleles compared with what one would expect for a lethal allele under mutation-selection balance or drift. Therefore, in cases with smaller numbers of recessive lethal loci with lower MAFs, the mortality caused by lethal alleles will be lower, subsequently resulting in a smaller gain achieved by distinguishing the two effects. However, several recessive lethal mutations have been identified in cattle breeds, and the numbers continue to increase (Cole 2015), with some reaching high recessive allele frequency (e.g., Kadri et al. 2014; Sahana et al. 2016; Hoff et al. 2017).

An additional assumption potentially prone to violation in real scenarios is the independence of recessive lethal loci and the independence of recessive lethal loci and nonlethal loci across the genome. In reality, recessive lethal alleles might be in LD with each other as well as with other loci. However, this is expected to have negligible consequences when using the novel approach that predicts risk probability due to lethal allele and polygenic effects separately, where polygenic GEBVs are estimated using Data_poly, but may cause confounding between the two effects when using the approach that does not distinguish the two effects, based on Data_all. An additional issue that was not taken into account in our simulation is the possibility of synergistic epistasis between the recessive lethal loci and other loci with polygenic effects. Under such interaction, the lethality, or penetrance, of recessive lethal loci may depend on polygenic effects, thus risking double counting of lethal effects when GEBVs are estimated in the presence of the lethal alleles. Such epistatic interactions were not considered in this study due to the complexity and lack of prior information for the simulation.

### Comparison of models

Categorical traits are not normally distributed, and thus linear mixed models are believed to behave poorly in modeling such traits (Portnoy 1982). Despite such violations of normality assumptions, the use of linear mixed models in the genetic analysis of categorical traits is gaining popularity due to their straightforward implementation. Meijering and Gianola (1985) demonstrated that LM can be applied without much loss of statistical power. In our study, slight differences in prediction accuracy were observed between the three models implemented, i.e., logit model, probit model, and LM, but the differences were not statistically significant. These results indicate that our approach can be implemented in LM with negligible loss of accuracy.

The regression coefficients of observed proportions of calf mortality against predicted risk probabilities were different from 1 when using the models that did not distinguish polygenic and lethal allele effects. The deviation from 1 was much larger for the LM than for the logit and probit models. This could partly be because in the LM, the threshold is approximated by direct calculation of mortality from the data, as opposed to the probit model, where the threshold is set to 0 for convenience and the underlying liability moves the origin accordingly. For the LM, the deviation from 1 was even larger for the approach based on Data_all that did not distinguish recessive lethal allele and polygenic effects. This could be explained by the fact that the threshold was approximated from observed mortality in the data, including mortality due to recessive lethal alleles, and hence the approximate threshold could be far from the threshold for the polygenic model. Moreover, the relationship between sire risk probability due to polygenic effects and offspring mortality is not necessarily linear. Consequently, the regression coefficient of observed mortality against the predicted risk probability might not necessarily be 1.

### Management of recessive lethal alleles in breeding programs

To date, commonly proposed methods for managing recessive lethal alleles have focused on the optimization of mate selection to avoid carrier-to-carrier matings. Van Eenennaam and Kinghorn (2014) proposed methods and programs that allow selection against the total number of lethal alleles and recessive lethal genotypes. Cole (2015) extended the parent-average penalizing method for controlling inbreeding proposed by Pryce et al. (2012), allowing it to consider information on recessive lethal alleles. Some studies also suggested the complete removal of carriers from the breeding population to eradicate recessive lethal mutations (e.g., Thompson et al. 2006).

Managing recessive lethal alleles requires a trade-off between controlling recessive lethal alleles in the long run and maintaining genetic gains in production and functional traits (Segelke et al. 2014). Previously proposed methods aimed at optimizing mate selection as well as culling carriers might allow the control of recessive lethal allele frequencies and avoid lethal homozygous genotypes. However, these methods represent classic tandem selection, where breeding animals are excluded from mating due to low merit for one trait (recessive lethal alleles in this case), regardless of their superiority in other traits. The consequence of this approach is a reduction in selection intensity and a subsequent reduction in genetic gain. The slightly different approach proposed by Segelke et al. (2014) recommends a selection index that weights the carrier status of recessive lethal haplotypes based on economic consequences and population allele frequencies when selecting females for mating. A drawback of this approach, and many other mate-allocation-based approaches, is the inability to handle many recessive lethal alleles. For instance, Cole (2015) pointed out the difficulty of assigning proper weights and costs for each recessive lethal allele as the number of identified alleles increases.

The approach proposed in this study enables blending polygenic breeding values for a given trait with risk probabilities from recessive lethal alleles. Thus, the method is beneficial for a balance between controlling recessive lethal frequencies in the population and maintaining genetic gains in economically important traits. In contrast to the methods where carrier status for each recessive lethal allele is a selection criterion, the proposed method integrates the effect of each recessive lethal allele into the breeding value for a particular trait (mortality or survival), which can be used for selection decisions. Therefore, an overall weight for the trait of interest can be used to integrate the breeding values, which account for both the polygenic and recessive lethal allele components, into a selection index with no need to assign weights for each recessive lethal allele.

## Conclusions

This study proposed an approach for predicting the probability of mortality of future offspring by predicting the risk probabilities from polygenic and recessive lethal components separately. The approach was tested using simulated data and found to be superior to approaches that do not distinguish polygenic and lethal allele effects. No statistically significant differences in prediction accuracy were observed between the probit model, logit model, and LM, suggesting that the novel approach can be implemented using different models, with comparable power.

## Data Availability

Data available from the Dryad Digital Repository: 10.5061/dryad.xd2547ddv.
